# Association of KDR rs1870377 genotype with clopidogrel resistance in patients with post percutaneous coronary intervention

**DOI:** 10.1016/j.heliyon.2021.e06251

**Published:** 2021-02-16

**Authors:** Wajdy Al Awaida, Ali A. Ahmed, Asia Ali Hamza, Khalid I. Amber, Hamzeh J. Al-Ameer, Yazun Jarrar, Ghizal Fatima, Ahmed O. Maslat, Yulia Gushchina, Omar Al bawareed, Najah R. Hadi

**Affiliations:** a Department of Biology and Biotechnology, American University of Madaba, Madaba 11821, Jordan; b Department of Pharmacology and Therapeutics, College of Medicine, University of Kufa, Iraq; c Department of Biochemistry, College of Medicine, University of Kufa, Iraq; d Al Najaf Center for Cardiovascular Surgery and Cardiac Catheterization in AL-Sadder Teaching Hospital in Al Najaf Al-Ashraf Governorate, Iraq; e Department of Pharmacy, College of Pharmacy, Alzaytoonah University of Jordan, 11734 Amman, Jordan; f Era's Medical College, Era University, Lucknow, India; g Department of Biological Sciences, Yarmouk University, Irbid, Jordan; h Department of General and Clinical Pharmacology, Рeoples’ Friendship University of Russia (RUDN University), 6 Miklukho-Maklaya Street, Moscow, 117198, Russian Federation; i Department of Normal Physiology, Рeoples’ Friendship University of Russia (RUDN University), 6 Miklukho-Maklaya Street, Moscow, 117198, Russian Federation

**Keywords:** *KDR*, Clopidogrel resistance, VEGFR2, Post percutaneous coronary intervention, SNP

## Abstract

**Background:**

Clopidogrel is an antiplatelet therapy that is widely used in pre and post percutaneous (PCI) coronary intervention procedures to prevent platelet aggregation and stent restenosis. However, there is a wide inter-individual variation in clopidogrel response and some patients showed resistance against the activity of Clopidogrel. Kinase insert domain receptor (*KDR)* gene is responsible for the transcription of vascular endothelial growth factor receptor 2 (VEGFR2) that plays a major role in the cardiovascular diseases (CVDs) and platelet aggregation. The aim of this study was to find out the association of *KDR rs1870377* genotype with clopidogrel resistance (CR) in CVD patients, of Iraqi Arabic origin, hospitalized for elective PCI.

**Materials and methods:**

This study was a case-control study with a total of 324 PCI patients. Those patients were classified into 213 patients with non-clopidogrel resistant and 111 patients with CR, depending on the analysis of platelet activity phenotype after clopidogrel administration. *KDR rs1870377* was genotyped for all patients using polymerase chain reaction-restriction fragment length polymorphism technique and confirmed by DNA Sänger sequencing through applying Biosystems Model (ABI3730x1).

**Results:**

*KDR rs1870377* SNP is strongly associated (Chi-sqaure, *p* vale <0.05) with CR under dominant, co-dominant and recessive models. Additionally, A allele in the *rs1870377* SNP may have an impact on the serum levels of VEGFR2 and low density lipoprotein.

**Conclusions:**

*KDR rs1870377* SNP is a potential genetic biomarker of CR among CVD patients of Iraqi Arabic origin. Further clinical studies, with larger sample, are required to confirm the findings of this study.

## 1 Introduction

Clopidogrel exhibits significant variability in its response ranging from over activity that may cause bleeding to loss of function that causes significant adverse cardiovascular events [1]. Although clopidogrel is still the most common irreversible antagonist of adenine diphosphate receptor used to inhibit platelet aggregation in percutaneous coronary intervention (PCI) and cardiovascular disease (CAD) patients [2], it has been reported that the main reason for the failure of PCI is the formation of platelet aggregation despite the use of clopidogrel in the treatment regimen [3]. The loss of the clopidogrel action phenomenon is known as clopidogrel resistance (CR), and this issue is attributed mainly to drug interaction, some diseases as diabetes mellitus, and genetic variants in genes related to the kinetics and dynamics of clopidogrel [4,5].

Recent studies clarified the strong association of atherosclerosis and the occurrence of CAD with a genetic variant in the *Kinase Insert Domain Receptor* (*KDR*) gene, specifically *the rs1870377* variant that responsible for transcription of vascular endothelial growth factor 2 (VEGFR2) receptor in vascular endothelial cells [6]. The VEGFR2 receptor plays a significant role in atherogenesis and platelet aggregation [7,8]. *KDR* gene is located on chromosome 4q12. The VEGFR2 receptor consists of 1356 amino acids, and the KDR gene splicing results in the formation of the VEGFR2 receptor with 679 amino acids that inhibit lymphatic blood vessel formation [9]. A nucleotide substitute of thymine (T) at the 1719 position on exon 11, by adenine (A), results in a variant (*rs1870377*) due to a missense mutation followed by an amino acid substitution. Such a substitution causes a dysfunction of the VEGFR2 receptor [10]. It has recently been reported that KDR *rs1870377* genetic variant is associated with CR among Chinese CVD patients [8].

Although there are reported studies regarding the genetic influence on CR among the different ethnic populations, there is no study investigating the effect of the KDR gene's genetic variants on clopidogrel response among Iraqi CVD patients. Accordingly, the present study aims to find out the association of *KDR rs1870377* genetic variant with CR among Iraqi CVD patients, of Arabic origin, on post percutaneous coronary intervention procedure.

## 2 Materials and Methods

### 2.1 Study population

The recent study is a case-control study. It comprises 324 CVD patients, which statistically represents the CVD patients on clopidogrel administration in Iraq. The inclusion criteria included that all participants were diagnosed as CAD patients with a need for PCI procedure. All of the volunteers were of Arabic origin. Non Arabic patients as Kurdish, Turkish, and Iranian were excluded from this study. The age of the patients ranged from 30 to 70 years.

The exclusion criteria included any patient with other chronic diseases, such as heart failure, hepatic and renal impairment, any recent hemorrhage, any recent surgical intervention within one month before PCI, any allergy to clopidogrel, heparin, or contrast media, in addition to patients that did not change the proton pump inhibitors (PPI) from omeprazole or esomeprazole into pantoprazole, which is known with the lowest drug-drug interaction with clopidogrel [11,12].

In AL-Sadder Teaching Hospital, specialist physicians from Al Najaf Center for cardiovascular surgery and cardiac catheterization in Al Najaf Al-Ashraf governorate documented that all participants selected from the same center met the criteria of this study.

Preparation of all participants started at least one week before PCI operation, according to the following steps:

1 Omeprazole and esomeprazole were replaced by pantoprazole [13].
2 Hospital admission was made at least 24 h before the PCR procedure.
3 Taking regular dual antiplatelet doses of 100 mg acetyl salicylic acid and 75 mg clopidogrel [14].
4 The loading dose for clopidogrel (600 mg) was administrated within the last 12–14 h in divided doses, two tablets every 2 h, before the PCI procedure [15].

The study was conducted following the Declaration of Helsinki. The Ethical Committee approved the study of Clinical studies at the Faculty of Medicine-University of Kufa. (No. MC09). All screened subjects gave signed informed consent before study activities.

### 2.2 Phenotypic classification of clopidogrel resistance

The two study groups' classification, the CR group and the non-clopidogrel resistant (NCR) group, were made depending on the ADP-induced platelet aggregation results. The patient was considered resistant to clopidogrel when the patient's platelet aggregation, on clopidogrel treatment, is more than 70% after adding ten μM ADP agonist [16]. Accordingly, the CR group included all patients with a loss of clopidogrel function that consisted of 111 patients, 37 females, and 74 males, with an age range of 55.82 ± 9.31. The NCR group included 213 CAD patients subjected to PCI that responded to clopidogrel as an inhibitor of platelet aggregation. The NCR group patients were 54 females and 159 males with an average age of 57.67 ± 7.99.

### 2.3 Blood analysis

In the morning and before subjecting for PCI procedure, a sample of five milliliters of venous blood was withdrawn from each patient. The blood sample was divided into three parts, 2 ml placed in an EDTA tube for DNA extraction, 1ml in a lithium-heparin tube for platelet aggregation test using Multiplate® analyzer from Roche company, and the last 2 ml were placed in a straight tube for serum VEGFR2 and other parameters [17]. The ELISA technique used to detect serum VEGFR2 through RayBio® human VEGFR2 ELISA kit. Additionally, estimation of body mass index (BMI) through equation formula for BMI which is weight in kilograms divided by height in meters squared while serum lipid profile levels including low-density lipoprotein (LDL), high-density lipoprotein (HDL), triglyceride (TG), cholesterol, and very-low-density lipoprotein (VLDL) were analyzed automatically in the hospital for all of the participants in this study using BIOLABO kits following manufacturer instructions. Regarding platelet aggregation test, it was performed using MULTIPLATE® analyzer by Roche company utilizing the ADP specific test with its reagents.

### 2.4 Genotype determination

DNA extraction was performed using a specific kit for DNA purification (Promega, USA). The protocol described by the manufacturer was followed.

The determination of *the KDR rs1870377* genetic variant was done using polymerase chain reaction-restriction fragment length polymorphism (PCR-RFLP) technique. The amplification of a DNA sequence containing *the KDR rs1870377* genetic variant was done using specific primers (Promega, USA). The sequence of forwarding primer is '5-TGCAAGTCCTCCACACTTCTCCAT-3,' and the reverse primer is '5-AAGGAGGCCAGTGGCTTCTAAGTT-3′, and the PCR consisted 35 cycles of denaturation at 95 °C for 1 min, primer annealing at 63 °C for 1 min and lately extension at 72 °C for 1 min, as described previously [18]. According to the manufacturer protocol, the PCR products were digested by specific restriction enzyme AluI (Promega, USA). The restricted PCR products were electrophoresis through 3% agarose gel. The genotyping results were confirmed through DNA Sanger sequencing by Applied Biosystems Model (ABI3730x1) (Macrogen, South Korea).

### 2.5 Statistical analysis

Continuous variables were illustrated by mean ± SD. Student's t-test to express the means variance between NCR and CR. ANOVA test was applied for describing level rates of continuous parameters in genotypes Through the SPSS v. 25.0 software (Chicago, IL SPSS Inc). Genotype distribution and allele frequency expression done by non-numerical variables. chi-squared test to assess the existence of differences of these variables. If p value was <0.05, then variations are considered significant.

#### 2.5.1 Logistic regression (Multinomial)

Logical regression was obtained by SPSS software, to predict the relevance of allele frequencies and genotype to CR with various models of inheritance. The rs1870377 SNP of KDR gene.

Odds ratio (OR) is the expression for the results regarding dissection for allele frequencies and genotype allocation, P-value and confidence interval (CI - 95%). Outcome adjustment for sex, age, BMI, HT, DM and smoking, OR, CI 95% and P-values were also estimated.

## 3 Results

After clopidogrel administration, the platelet aggregation results revealed that 111 patients, out of 324, (34.26%) have CR. Table 1 shows the demographic, lipid profile, and platelet activity of the patients. There is no statistically significant difference (*p*-value > 0.06) in the sex, age, body mass index, platelet account, heart failure and diabetes frequency, smoking, VLDL, HDL, and TG between the CR and NCR patients. Additionally, we did not find a statistical difference (*p*-value = 0.778) in the VEGFR2 serum levels between CR and NCR groups. However, we found a significant difference in the cholesterol (*p*-value = 0.023) and LDL (*p*-value = 0.033) serum levels between CR and NCR patients (Table 1).

**Table 1:** Base line characteristic parameters (Anthropometric, biochemical and environmental) for the study participants.

Parameter	NCR (control)	CR(disease)	*P*-value
No. (male/female)	213 (159/54)	111 (74/37)	0.13
Age	57.67 ± 7.99	55.82 ± 9.31	0.062
BMI	28.71 ± 4.23	29.42 ± 4.74	0.170
Cholesterol (mg/dl)	288.00 ± 9.64	290.67 ± 10.75	0.023
TG	240.79 ± 18.63	238.51 ± 19.45	0.299
VLDL	48.15 ± 3.67	47.70 ± 3.89	0.305
LDL	207.39 ± 11.53	210.43 ± 13.20	0.033
HDL	32.45 ± 3.19	32.54 ± 3.46	0.815
Platelet count (×10^3^/mm^3^)	240.41 ± 49.62	249.21 ± 67.33	0.182
VEGFR (pg/ml)	8075 ± 687	8098 ± 731	0.779
Diseased vessels	157	90	0.569
HT	192	102	0.90
DM	119	60	0.86
Smoker	103	62	0.46
CCBs	37	22	0.653
ACEI/ARBs	51	35	0.268
B-Blockers	131	70	0.894
Diuretics	64	38	0.580
PPI	33	26	0.150
Nitrates	104	59	0.672

Abbreviations: CR, clopidogrel resistant; NCR, non-clopidogrel resistant; HT, hypertension; DM, diabetes mellitus; BMI, body mass index; LDL, low-density lipoprotein; HDL, high-density lipoprotein; TG, triglyceride, CCBs, calcium channel blockers; ACEI/ARBs, angiotensin-converting enzyme inhibitor/angiotensin receptor blocker; PPI, proton pump inhibitor.

Regarding the drugs used by the patients, there was no statistically significant difference (*p*-value = 0.150) in the frequency of used drugs between CR and NCR, as represented in Table 2.

**Table 2:** Drugs consumed by the patient participants.

Parameter	CR (111/324)	NCR (213/324)	P-value
CCBs	22	37	0.653
ACEI/ARBs	35	51	0.268
B-Blockers	70	131	0.894
Diuretics	38	64	0.580
PPI	26	33	0.150
Nitrates	59	104	0.672

Abbreviations: CCBs, calcium channel blockers; ACEI/ARBs, angiotensin-converting enzyme inhibitor/angiotensin receptor blocker; PPI, proton pump inhibitor.

### 3.1 *KDR rs1870377* genotyping

**Figure 1:**
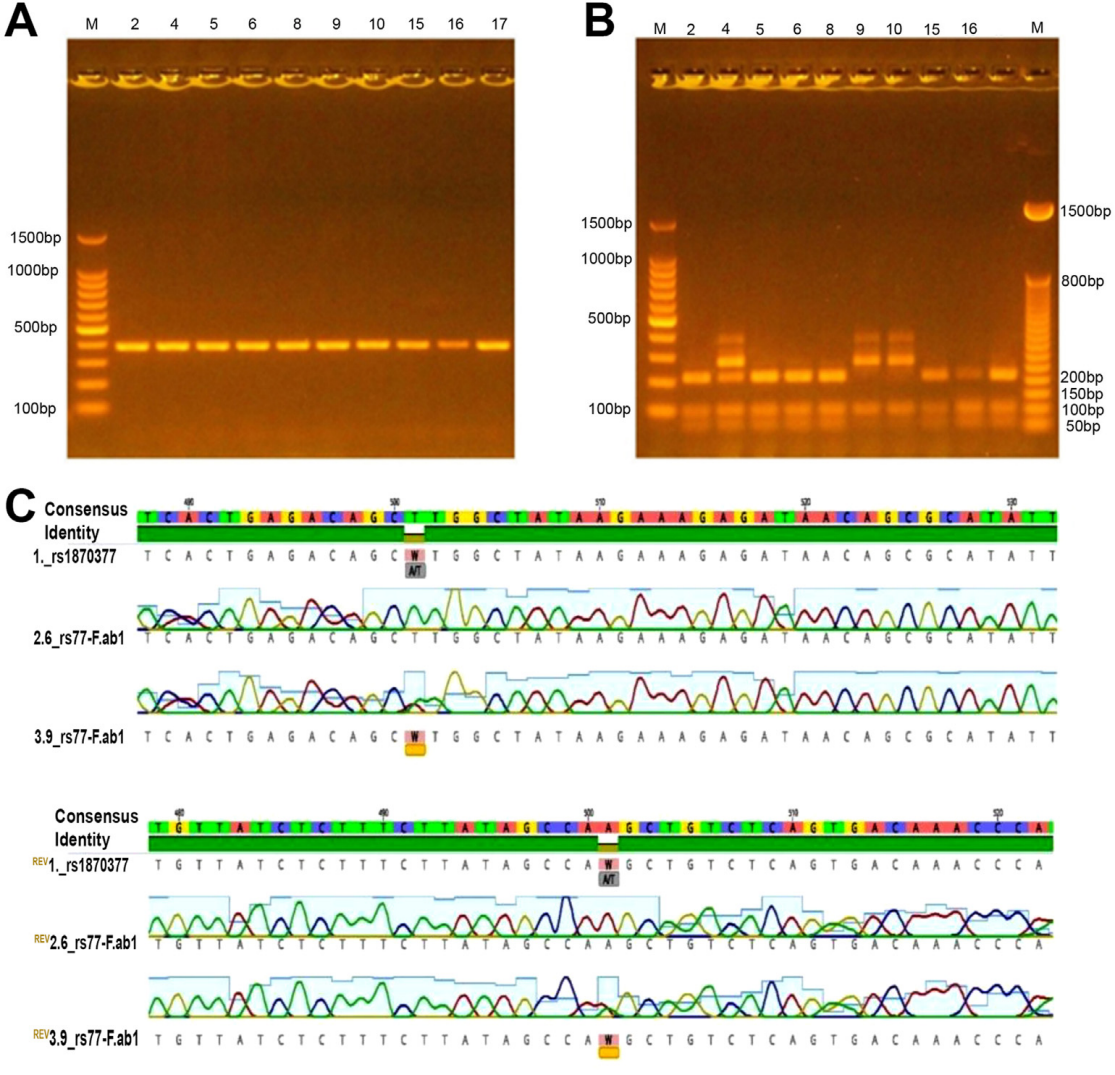
**A**: PCR product with 382 bp. **B**: Fragment of DNA for rs1870377, Lane number 9 and 10 represent the wild genotype, Lane number 4 represents the heterozygous genotype, Lanes number 2,5,6,8,15,16 and 17 describe the homozygous recessive genotype. Two ladders were used (100 bp and 50 bp) on both sides of the mold with the symbol M. **C**: Gene sequencing for documenting the RFLP results.

Figure 1A shows the 3% gel electrophoresis of the PCR product amplification of *the KDR* gene with a 382 bp size. Simultaneously, Figure 1B represents the *KDR rs1870377* genotype after restriction of the PCR products through *the AluI* enzyme. The wild *KDR rs1*870377 T/T genotype was represented by three bands, 104, 278, and 382 bp. Three bands with different sizes represented the homozygous *KDR rs1*870377 A/A genotype; 58, 104, and 220 bp. Lately, the *KDR rs1*870377 T/A heterozygous genotype was represented by five bands 58, 104, 220, 278, and 382 bp after gel electrophoresis. Additionally, the results were confirmed through DNA sequencing (Figure 1C). Our results showed that the frequency of the wild, heterozygous, and homozygous *KDR rs1870377* genotype among CR patients is 55.86%, 36.03%, and 8.11%, respectively. The wild, heterozygous, and homozygous *KDR rs1870377* genotype among NCR patients is 74.18%, 23.00%, and 2.82%. We found no deviation (X^2^ test, *p* > 0.362) from the Hardy-Weinberg equation of the frequency of *KDR rs1870377* genotypes among both CR and NCR patients.

### 3.2 Association between *KDR rs1870377* genotype and CR

The present study showed a significant association of *KDR rs1870377* allele and genotype frequency with CR using all inheritance models without adjustment and after adjustment with body mass index, smoking, diabetes, hypertension, age, and sex (Table 3).

**Table 3:** Association of *KDR rs1870377* genotype with CR occurrence.

Rs1870377 (T/A)	Control N = 213	CR N = 111	Unadjusted OR.	Unadjusted (95%CI)	P-value	Adjusted OR	Adjusted (95%CI)	P-value
Co-dominant								
TT(Ref.)	158 (74.18 %)	62 (55.86 %)						
TA	49 (23.00 %)	40 (36.03 %)	2.08	1.24–3.46	0.005	2.259	1.31–3.87	0.003
AA	6 (2.82 %)	9 (8.11 %)	3.82	1.30–11.18	0.000	4.329	1.44–12.96	0.009
Dominant								
AA + TA vs TT	55 (25.82 %)	49 (44.14 %)	2.27	1.39–3.68	0.001	2.48	1.48–4.15	0.001
Recessive								
TT + TA (Ref.)	207 (97.18 %)	102 (91.89 %)						
AA	6 (2.82 %)	9 (8.11 %)	3.02	1.05–8.74	0.04	3.25	1.10–9.54	0.032
Additive								
2(AA)+TA	61 (28.64 %)	58 (52.25 %)	2.83	1.76–4.55	0.0001			
MAF%(A)	14.32 %	26.13 %	1.82	1.19–2.79	0.005			

Using the co-dominant model, the heterozygous T/A shows a significant association with CR compared with the NCR group without adjustment (OR = 2.08, CI 95%; 1.24–3.46, *p* = 0.005) and with adjustment (OR = 2.25, CI 95%; 1.31–3.87, *p* = 0.003). The homozygous genotype A/A revealed a significant association with the occurrences of CR (OR = 3.82, CI 95%; 1.30–11.18, P < 0.001) before an adjustment and after adjustment for the studied parameters. The dominant model also showed that *KDR rs1870377* A allele have a significant effect on CR occurrence using the unadjusted model (OR = 2.27, CI 95%; 1.39–3.68, *p* = 0.001), as well as after adjustment of the studied parameters (OR = 2.48, CI 95%; 1.48–4.15, *p* = 0.001). The recessive model also showed a significant association of *KDR rs1*870377 A/A genotype with CR before adjustment (OR = 3.02, CI 95%; 1.05–8.74, *p* = 0.04), as well as after adjustment of the study parameters (OR = 3.25, CI 95%; 1.10–9.54, *p* = 0.03). Furthermore, the additives model showed a significant association of *KDR rs1870377* genotype with CR (OR = 2.83, CI 95%; 1.76–4.55, *p* = 0.0001) NCR group.

### 3.3 Association of *KDR rs1870377* genotype with lipid profile, serum VEGFR2 level, platelet count, and body mass index

Serum KDR level, serum lipids, BMI and platelet count were also tested against the dominant and the co-dominant genotype models as in Tables 4 and 5. The outcome results only clarify the significant correlation of LDL and serum KDR level with the (A) allele in both models (dominant and co-dominant) in CR patients with a *p* vale <0.05 (see Tables 4 and 5).

**Table 4:** Association of *KDR rs1870377* genotype with lipid profile, serum VEGFR2 level, platelet count and body mass index under the co-dominant model.

Clinical characteristic	TT (62 patients)	TA (40 patients)	AA (9 patients)	*P*-value
LDL (mg/dl)	213.83 ± 8.03	218.95 ± 12.99	226.22 ± 17.27	∗0.002
VLDL (mg/dl)	45.72 ± 1.60	45.17 ± 4.06	45.88 ± 3.82	0.606
HDL (mg/dl)	30.93 ± 2.45	31.10 ± 2.56	31 ± 2.44	0.944
TG (mg/dl)	228.06 ± 16.82	225.87 ± 20.34	234.44 ± 5.27	0.416
Cholesterol (mg/dl)	295.48 ± 9.98	293.62 ± 10.56	302.22 ± 14.38	0.092
BMI (kg/m^2^)	28.61 ± 4.14	28.38 ± 4.92	28.52 ± 4.77	0.968
Platelet count (×10^3^/mm^3^)	265.33 ± 22.74	256.85 ± 25.34	258.22 ± 20.25	0.190
VEGFR2 (pg/ml)	7590.48 ± 86.14	7998.75 ± 145.81	9180 ± 1012.5	∗0.000

∗ indicates statistical significance with *p*-value < 0.05.

**Table 5:** Association of *KDR rs1870377* genotype with lipid profile, serum VEGFR2 level, platelet count and body mass index under a dominant model.

Clinical characteristic	TT (62patients)	TA + AA (49 patients)	*P*-value
LDL (mg/dl)	213.83 ± 8.03	220.28 ± 13.96	∗0.0028
VLDL (mg/dl)	45.72 ± 1.60	45.30 ± 3.99	0.451
HDL (mg/dl)	30.93 ± 2.45	31.08 ± 2.51	0.752
TG (mg/dl)	228.06 ± 16.82	227.44 ± 18.76	0.854
Cholesterol (mg/dl)	295.48 ± 9.98	295.20 ± 11.67	0.891
BMI (kg/m^2^)	28.61 ± 4.14	28.41 ± 4.84	0.815
Platelet count (×10^3^/mm^3^)	265.33 ± 22.74	257.10 ± 24.30	0.069
VEGFR2 (pg/ml)	7590.48 ± 86.14	8215.71 ± 633.80	0.000

∗ indicates statistical significance with *p*-value < 0.05.

## 4 Discussion

Several studies reported the inter-individual variation in clopidogrel response among CVD patients [2,19,20]. The genetic factor plays a significant role in this inter-individual variation. It is well reported that *CYP2C19* genetic variants are a clinical biomarker for clopidogrel response [21]. However, other genetic variants on other genes may also play a role in this inter-individual variation in clopidogrel response and the occurrence of CR among CVD patients. The Iraqi population consists of different sub-ethnic Caucasian and Asian populations, such as Iranian, Kurdish, and Turkish. Since the ethnic variation in the frequency of genetic variants is reported previously [22,23] and the variation in drug response, it is recommended to confirm this study's finding among other sub-ethnic groups in Iraq and other Arabic groups living outside Iraq. This study showed that *the KDR rs1870377* genotype is strongly associated with CR among CVD patients of Iraqi Arabic origin on post percutaneous coronary intervention procedure. This finding may increase our understanding of the *KDR* gene's role and its genotype in clopidogrel response among CVD patients on clopidogrel administration. Further clinical studies are needed to confirm this finding among Iraqi CVD patients.

It has been found that the *CYP2C19* genotype is associated with clopidogrel response and CR among Iraqi patients after PCI [21]. Our study added that, in addition to *the CYP2C19* genotype, the *rs1870377* SNP *in KDR* gene can be considered as genetic biomarker for clopidogrel non-responsiveness, so that the risk of platelet aggregation and its consequence CVD complication are reduced [24].

Furthermore, present study illustrate that A allele in the *rs1*870377 has a significant correlation with LDL levels among dominant and co-dominant pattern. This may indicate that increased LDL levels at least in part, has an impact role in reducing the response of CVD patients toward clopidogrel. It has been reported that LDL and cholesterol levels are correlated significantly with increased platelet aggregation [25]. Additionally, findings of the present study are in line with what was reported previously by Uzun et al. (2015) that LDL and cholesterol levels were higher among CR than NCR patients [26]. These findings may indicate that high LDL levels and cholesterol have a strong impact on CR among CVD patients.

Moreover, this study showed that the *KDR rs1870377* genotype frequency is similar to what was reported among other Caucasian populations but different signs than those reported among Asians [27,28,29]. Accordingly, this finding may play a part in the inter-ethnic variation in clopidogrel response and the prevalence of CR among different ethnic groups [30,31,32,33].

Zhang et al. studied the effect of *the KDR rs1870377* genotype on clopidogrel response among Chines patients, but he did not find an association of the *KDR rs1870377* genotype with CR [8]. However, other studies demonstrated the relevance of this genetic variant with CAD [6,34]. Our investigation showed that *KDR rs1870377* A allele is associated significantly with CR in the dominant, co-dominant, and recessive models. Even before and after adjustment with other environmental factors, such as body mass index, hypertension, and age. Therefore, these results indicate a strong association between *the KDR rs1870377* SNP and CR. The A allele in *KDR rs1870377* also has a significant correlation with serum VEGFR2 in both dominant and co-dominant pattern which indicates that *KDR rs1870377* A variant causes an alteration in the serum level of VEGFR2 and hence affecting on clopidogrel response and this may be due to production of non-functional VEGFR2 receptors as the serum VEGFR2 are soluble receptors results from splicing of KDR gene responsible for expression of cell surface VEGFR2 receptors [9].

There are some limitations to this study. First: We did not study other genetic variants in *the KDR* gene, which may have an additive effect with the *rs1870377* variant on clopidogrel response. Second: Other sub-ethnic groups in Iraq were not included in this study. Lastly, other factors that may influence clopidogrel response, such as medication adherence, were not studied.

## 5 Conclusions

The rs1870377SNP in KDR gene is strongly associated with Clopidogrel Resistance among CVD patients of Iraqi Arabic origin and could be considered as a potential biomarker for this phenomenon. However, further clinical studies are needed to confirm this finding among other ethnic groups.

## Declarations

### Author contribution statement

Wajdy Al Awaida: Conceived and designed the experiments; Performed the experiments; Analyzed and interpreted the data; Wrote the paper.

Ali A Ahmed: Conceived and designed the experiments; Performed the experiments; Analyzed and interpreted the data; Contributed reagents, materials, analysis tools or data; Wrote the paper.

Najah R Hadi: Conceived and designed the experiments; Contributed reagents, materials, analysis tools or data; Wrote the paper.

Asia Ali Hamza: Conceived and designed the experiments; Contributed reagents, materials, analysis tools or data.

Khalid I Amber: Performed the experiments; Analyzed and interpreted the data; Contributed reagents, materials, analysis tools or data.

Hamzeh J. Al-Ameer; Ahmed O Maslat; Yulia Gushchina; Omar Al bawareed: Performed the experiments; Analyzed and interpreted the data.

Ghizal Fatima: Performed the experiments; Wrote the paper.

Yazun Jarrar: Contributed reagents, materials, analysis tools or data; Wrote the paper.

### Funding statement

This work was supported by the Рeoples’ Friendship University of Russia (RUDN University) Strategic Academic Leadership Program.

### Data availability statement

Data will be made available on request.

### Declaration of interests statement

The authors declare no conflict of interest.

### Additional information

No additional information is available for this paper.
